# Supplementary material for: Management of depression in people living with HIV/AIDS in Senegal: Acceptability, feasibility and benefits of group interpersonal therapy
Source: Glob Ment Health (Camb). 2023 Jun 30;10:e36. doi: 10.1017/gmh.2023.31 (PMC10579691; doi:10.1017/gmh.2023.31)

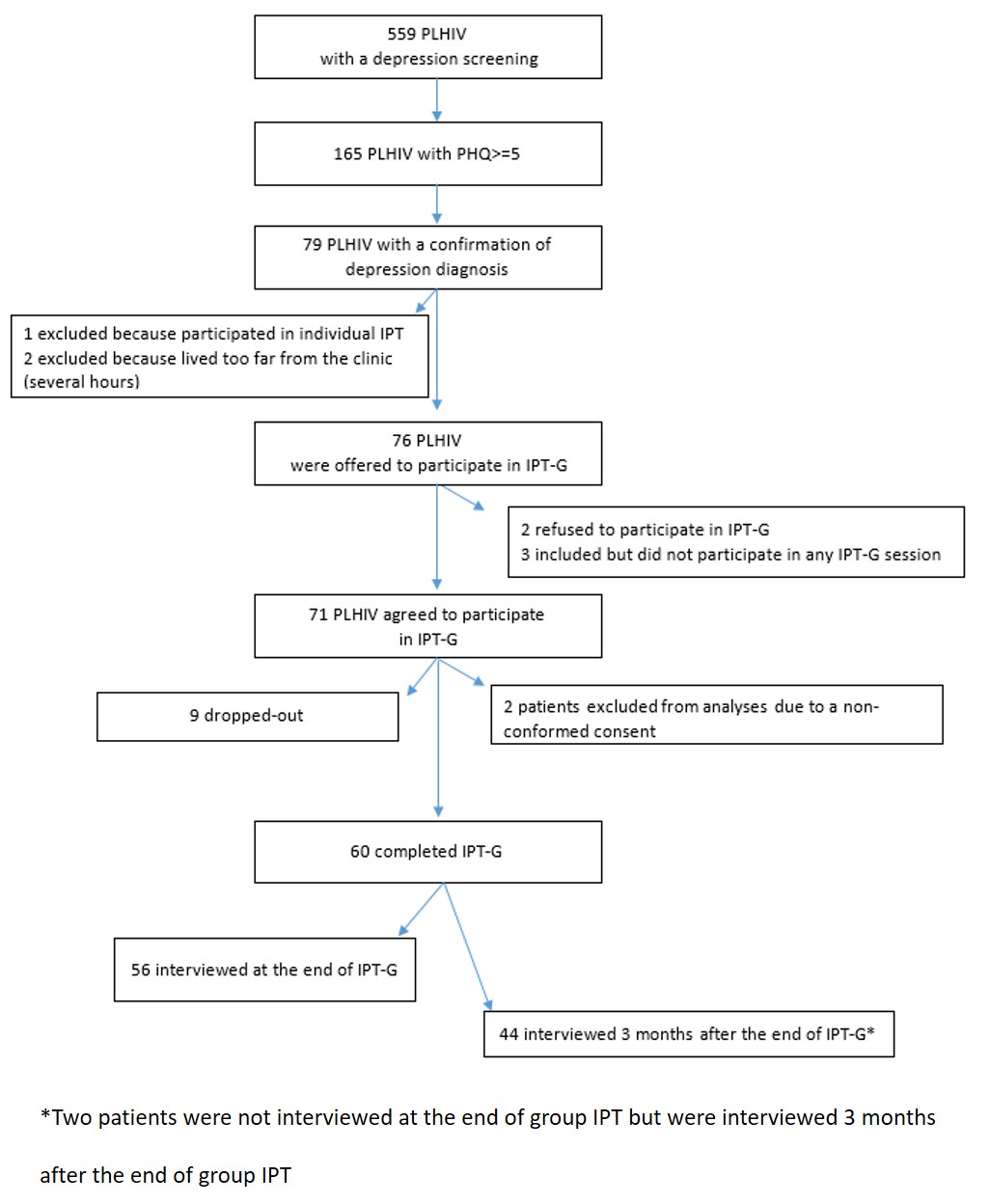


**Figure S1: Flow Chart of study participants**

Table S1: Characteristics of the patients who dropped out and the patients who completed group IPT

| Variables | Patients who complete group IPT | Patients who dropped out | p-value |  |  |
| --- | --- | --- | --- | --- | --- |
| N | 60 | 9 |  |  |  |
| Age (years) (median IQR) | 41 (49-55) | 42 (47-54) | 0.85 |  |  |
| Female | 29 (48%) | 6 (67%) | 0.48 |  |  |
| Living alone | 26 (43%) | 4 (44%) | 1.00 |  |  |
| Unemployed | 24 (40%) | 5 (56%) | 0.48 |  |  |
| No Sharing status (mis. 2) | 11 (18%) | 3 (33%) | 0.66 |  |  |
| Financial difficulties | 49 (82%) | 8 (89%) | 1.00 |  |  |
| PHQ-9 total score | 13 (10-15) | 13 (12-14) | 0.98 |  |  |

Figure S2: Assessment of the feasibility by the facilitators (N=3)


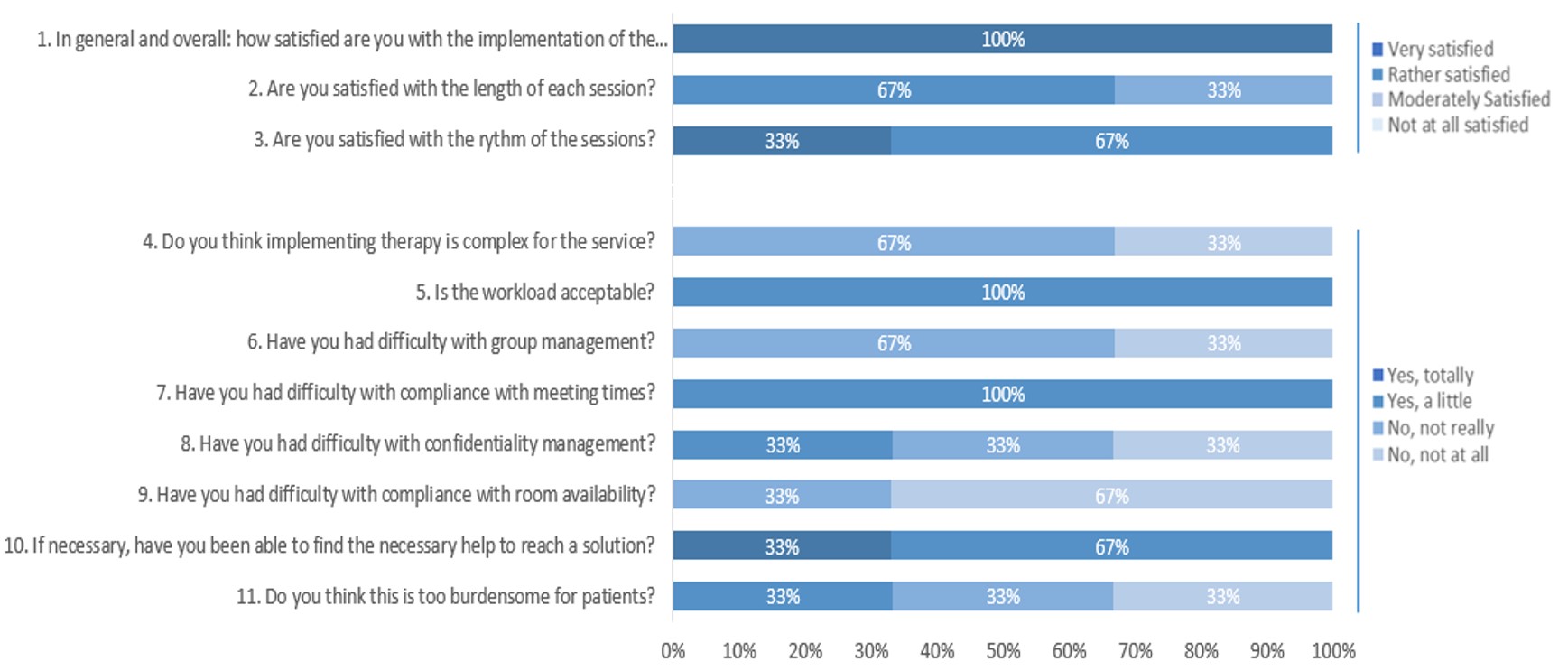

Supplement: Supplementary file 1 [file S2054425123000316sup001.docx]
